# Supplementary material for: Tead1a Initiates Transcriptional Priming Through the TEAD1a/YAP‐Notch1‐Spi1/Cebpα Axis to Promote Neutrophil Fate
Source: Adv Sci (Weinh). 2025 Aug 28;12(41):e05441. doi: 10.1002/advs.202505441 (PMC12591127; doi:10.1002/advs.202505441)
Supplement: Supplementary file 4 — Supplementary Dataset [file ADVS-12-e05441-s004.zip › 3-2.zip!/3-2/02-2.cluster/cluster5/Enrich/ego_CC.pdf]

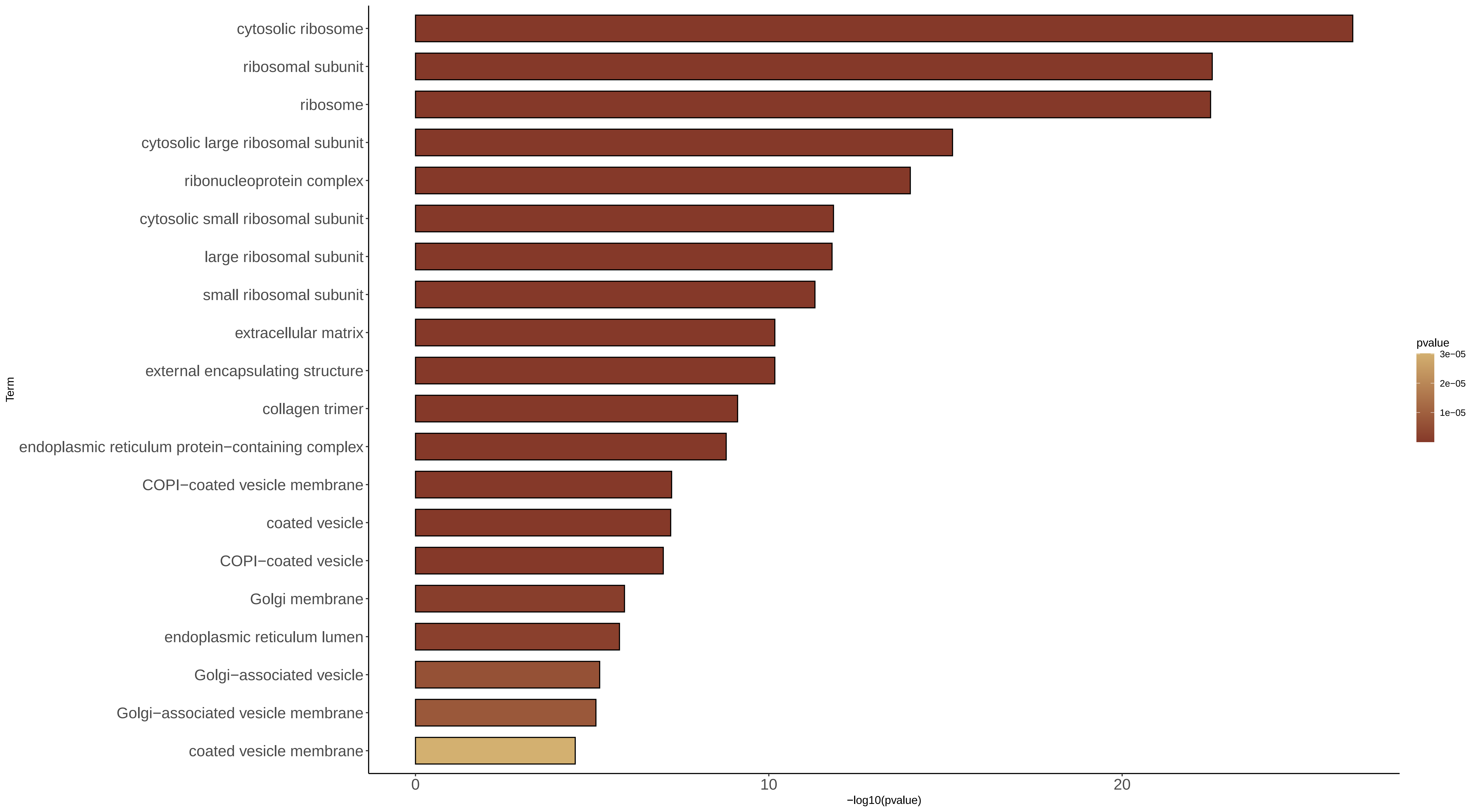
cytosolic ribosome
ribosomal subunit
ribosome
cytosolic large ribosomal subunit
ribonucleoprotein complex
cytosolic small ribosomal subunit
large ribosomal subunit
small ribosomal subunit
extracellular matrix
external encapsulating structure
collagen trimer
endoplasmic reticulum protein−containing complex
COPI−coated vesicle membrane
coated vesicle
COPI−coated vesicle
Golgi membrane
endoplasmic reticulum lumen
Golgi−associated vesicle
Golgi−associated vesicle membrane
coated vesicle membrane
Term
0
10
20
−log10(pvalue)
pvalue
3e−05
2e−05
1e−05
